# Pathology and Semiology

**Published:** 1880-07

**Authors:** D. C. Hawxhurst


### PATHOLOGY AND SEMIOLOGY.

BY D. C. HAWXHURST. D. D. S.

The subject of pathology and semiology seem to be very appropriately placed together, and are to be reported upon by the same committee. They naturally go together, because the point at which the dentist touches his patient is in treatment. Before he can treat he must know the pathology of his case, and he knows this by *symptoms*. Thus pathology and semiology go together, the former springing out of the latter.

Pathology (abnormal action) begins in cells. A study of pathology involves a large use of the microscope, of course. When we say that abnormal action begins in cells we recognize that the activities of the cell are very greatly under the control of the nervous system, and that changes in the nervous system, from emotion and so on, modify that action in the cells; and that cellular action is also under the control, to a certain degree, of influence, affecting the peripheral as well as the general nervous system, as heat and cold, and wet and dry, etc. But whatever the controlling cause, the action is in the cell, and thus it comes to be a question of cell action. Virchow recognized *that*; when he wrote his work on cellular pathology so many years ago, the work marked a great advance in the development of the subject. Before that we had no pathology that was worth anything. It was in 1836–7 that Schwann carried the doctrine of the cell from vegetable physiology into animal physiology, and soon after that Virchow began that remarkable course of investigation, which resulted in giving us a cellular pathology as a guide in our studies of disease.

Of course the normal phenomena of life are very various, and as we see them with the eye unaided they are inexplicable; but the phenomena of life, as manifested in the cell, are susceptable of analysis. Here alone can there be any approach to an understanding of their nature. To studies of the cell we must return as a point of departure for pathology, thus it is in certain changes from normal action, which go on in a morphological element too small to be seen by the naked eye, that we must find not only our physiology but also our pathology.

The phenomena of life we may name as nutrition, reproduction, motion, and so on ; these are to be seen in the cells, and may be best studied perhaps in the white blood corpuscles, in the amœba, or in any of the unicellular animalculæ. The normal phenomena of life are the basis of our comprehension of all *deviations* from health. Thus it happens that we are obliged to spend months and years in the study of the phenomena of cell action, normally carried on, before we can comprehend those deviations which differ but little from the normal, yet enough to constitute disease ; only thus can we become pathologists in the highest sense. There are very few excellent pathologists, for it requires more than a common medical course to make a man a thorough pathologist; this is not an easy task in the present state of medical education in this country and in England. It is necessary for us to begin our pathological studies in true earnest, after we have passed through such courses in medicine and dentistry as are possible in this country.

I have said that any deviation from normal cellular nutrition is to be looked upon in the light of pathology, as being abnormal and as either constituting disease or leading to it, as being, in fact, the *beginning* of disease. In studying this subject we should take account of the pabulum which supplies the cells, because the cell has to eat every day, or constantly, as certainly as the man ; the one is the thing in little and the other is the thing in large. The pabulum of cells, then, is worthy of some attention, and it must be necessary to name the three classes of nutriment, the three classes of proximate principles which are to be furnished constantly to the cell—in brief, the proteids, fats and salts. These must be furnished to every cell, because the part of the cell in which activity takes place, the germinal matter, has always the same chemical constitution and must always contain these three proximate principles, or its activities will cease.

Now the molecular changes which go on in the cell proceed because the conditions necessary for these changes are present, and when these conditions are destroyed through the lack of sufficient heat or of a proper supply of nutriment, or a proper amount of diluting fluid, or through the lack of some mineral or chemical elements which should exist, they either decline or are accelerated, or they deviate from their natural character qualitatively. In the latter case abnormal peculiarity of action ushers in disease.

Cells, of course, excrete matter; they take in new matter; they convert this pabulum into their own substance, and there are cell circulation, imbibition, endosmosis and exosmosis. I merely mention these activities as a reminder that we should ever give our most careful attention to pathology from this inmost standpoint, for there is no plainer truth than that whatever of success we attain to in the cure of disease must be through treatment directed to the modification of life processes as displayed in the activities of cells.

The work of cells (independently of their own changes, whereby their constitution is kept normal,) is to make tissue, to build nerve and muscular fibres, to weave the elastic tissue of the system, to lay down bone, to make cartilage, to make tooth structure, to make the nail, the hair, the skin, to construct the vascular tubes that carry the blood. The precise methods by which the cell produces these tissues need not here be stated. I know of no work that is better calculated to give a knowledge of the subject than that of Todd, Bowman and Beale, published, I think, in 1871. I have intended only to call attention to the much neglected basis of all rational knowledge of pathology, not to give an exposition of the principles of pathology here. These minute studies are demanded from *a priori* consideration and must here be insisted upon.

If we have not been able to make them as we should have done, we may repair the neglect by giving our *students* correct advice in this matter, and sending them on with the expectation of spending sufficient time and making sufficient effort to master the subject.

Of course we may treat disease, being guided by its superficial aspects, and learn to recognize it by its symptoms. We may thus know often what to do, and do it well. In many cases, however, we must do our duty, but blindly if we are unacquainted with what we may term microscopic pathology. At the very time when we most need its diagnostic and practical assistance, we shall be at fault.

It is true that we recognize disease by symptoms, but we know what it is in great measure, through the studies which I am describing. We cannot always enter into the study of cells, when our patient is before us—sometimes we can—sometimes we can take the blood, or a little portion of the tissue, or we can examine a tooth which we have been obliged to extract; we can study secretions or excretions—but it is not usually so. In general we recognize the disease by a study of symptoms. If we are cellular pathologists we shall often be able to judge of the cell activities that are going on by these signs, and be better able to adopt remedial measures, and to institute such cell changes in the direction of a more normal condition as may ultimately bring about health. In every case we shall be far more likely to understand our case if we are well grounded in cellular pathology.

I know those who will tell you that such an understanding of disease is impossible without it. They will tell that it is disgraceful to be ignorant of the labors and investigations of Virchow, Beale, Reindfleisch and the other great pathologists. Whatever we may say of this strong language, let us remember that it has been spoken.

The literature of the second department of my subject is very deficient. We have no good dental work on symptoms and *deferential diagnosis.* Somebody ought to write this work. It should be concise and full of points, and perhaps should not attempt to take up treatment; or, it might embody hints on treatment, incidentally.

But to enable the dentist to diagnose the condition and distinguish the disease, should be its main end. This has proved a great end and one difficult to reach in medicine. We need a work that shall perform the same function in dentistry. It is often difficult to distinguish one pathological state from another. The patient comes with pain; we do not know what that pain is caused by; sometimes we can tell at once, but often we cannot. For instance, we cannot always tell whether we have a case of pulp calcification, or a case of simple pulpitis, or a case of gout in the pulp, of rheumatism in the pulp, or a case of exostosis, in which the new formation about the foramen encroaches upon the nerves that enter it, or whether it is a case of simple neuralgia. In many a case the practitioner is is not able to distinguish, and those neighboring practitioners whom he has consulted in regard to it were not able to distinguish. In the Ohio Dental Association a number of years ago, the question was raised how to diagnosticate a case of pulp calcification from the other affections which it resembles in its manifestation of symptoms. That the greater number of members present were lacking in the means for perfecting that diagnosis must at least show that there are difficult pathological problems even within the limits of a tooth, leaving contiguous parts, and their pathological states, out of the question. It is extremely difficult to do it. At the same time there are some indications that look in the right direction, and which the old practitioner will recognize; but the inexperienced practitioner needs a work which shall at least put him in possession of what is known upon the subject.

An advancing wisdom tooth may produce certain painful symptoms which it is very difficult to distinguish from abscess, from pulpitis, or from various other affections that in their first stages all seem to be, so far as their symptoms go, very nearly alike. Such diagnostic points as there are to aid the student and the practitioner in the more intricate and obscure portion of his daily duties should be laid down in some systematic work, accessible to all, instead of being scattered as now through many journals and books.

Perhaps no disease is more protean in its form than irritation of the spine, which may cause pain in the mouth about the teeth, jaws, or in the temples, and which is often wholly misapprehended and mistreated, and is so misinterpreted by both dentist and doctor. It is a disease which in reality will give way immediately to appropriate treatment applied to the spine, or to the cause if that be not in the spinal axis. Even so apparently simple a thing as sensitiveness of the dentine may be due to constitutional causes, and may not be easily controlled except through systemic remedies. It is important to know if it be due to general acidity of the secretions, or whether it is a simple case of menstruation with irritation in the region of the teeth or what it is.

In syphilitic pains in the jaws we have another question which should be studied carefully by the dentist, and which should be treated of in such a work. These pains may resemble neuralgia of local origin, or a variety of other affections. A man may come with the gout and nothing else; if this should be mistaken for something wrong about the teeth—pulpitis or periostitis simple—and the tooth should be extracted, it would be a very bad case for the dentist. It is not a long time since I became acquainted with a case in which the pain passed instantly from the tooth to the toe, and the man when his system was inquired into was found to be subject to gout; the pain went back to his tooth which was perfectly sound; it seemed to be a clear case of gout. A great many cases of this kind and many cases of neuralgia could be brought into a work of this kind; they need to be carefully distinguished and each particular kind described.

The question of tumors should be thoroughly treated and each one distinguished from its congeners, and other affections also. There are many diseases connected with the antrum which afford a wide field for diagnostic description.

I hope some one will take upon himself this task. It is something that our profession needs now. It has now arrived at a point where it could appreciate a work of this kind—at a point indeed at which it is capable of producing such a work.

The questions of pathology and semiology are in a very prosperous condition; the amount of subject matter that has been accumulated in the journals and books is great, and all that is needed is that somebody should gather it up and put it into shape.

